# The duration of hypothermia affects short-term neuroprotection in a mouse model of neonatal hypoxic ischaemic injury

**DOI:** 10.1371/journal.pone.0199890

**Published:** 2018-07-03

**Authors:** Eridan Rocha-Ferreira, Amy Vincent, Sarah Bright, Donald M. Peebles, Mariya Hristova

**Affiliations:** UCL Institute for Women’s Health, Maternal & Fetal Medicine, Perinatal Brain Repair Group, London, United Kingdom; Hopital Robert Debre, FRANCE

## Abstract

Neonatal hypoxic-ischaemic encephalopathy (HIE) is major cause of neonatal mortality and morbidity. Therapeutic hypothermia is standard clinical care for moderate hypoxic-ischaemic (HI) brain injury, however it reduces the risk of death and disability only by 11% and 40% of the treated infants still develop disabilities. Thus it is necessary to develop supplementary therapies to complement therapeutic hypothermia in the treatment of neonatal HIE. The modified Rice-Vannucci model of HI in the neonatal mouse is well developed and widely applied with different periods of hypothermia used as neuroprotective strategy in combination with other agents. However, different studies use different periods, time of initiation and duration of hypothermia following HI, with subsequent varying degrees of neuroprotection. So far most rodent data is obtained using exposure to 5-6h of therapeutic hypothermia. Our aim was to compare the effect of exposure to three different short periods of hypothermia (1h, 1.5h and 2h) following HI insult in the postnatal day 7 C57/Bl6 mouse, and to determine the shortest period providing neuroprotection. Our data suggests that 1h and 1.5h of hypothermia delayed by 20min following a 60min exposure to 8%O_2_ do not prove neuroprotective. However, 2h of hypothermia significantly reduced tissue loss, TUNEL+ cell death and microglia and astroglia activation. We also observed improved functional outcome 7 days after HI. We suggest that the minimal period of cooling necessary to provide moderate short term neuroprotection and appropriate for the development and testing of combined treatment is 2h.

## Introduction

Neonatal hypoxic-ischaemic encephalopathy (HIE) affects 1–3 per 1000 live term births in high-income countries, and is a major cause of neonatal mortality and morbidity, with lifelong incapacitating consequences like cerebral palsy, epilepsy and mental retardation [1]. Birth asphyxia causes an annual estimate of one million or 23% of all neonatal deaths worldwide [2,3]. Therapeutic hypothermia has been introduced as standard clinical care for moderate to severe neonatal hypoxic-ischaemic (HI) brain injury [4,5], however it reduces the risk of death and disability only by 11% with about 40% of the treated infants still developing neurological disabilities [6]. Therefore it is necessary to develop simple, safe and effective supplementary therapies to add to the current therapeutic strategy in infants with HIE. The rodent model of neonatal HI has been well characterized [7] and studies have used different therapeutic hypothermia duration (3-6h) as a means for neuroprotection [8–10]. Animal studies have looked into further increase in duration of hypothermia, where in the preterm sheep model an increase from 72h to 120h of hypothermia does not prove protective and is deleterious in some brain regions [11]. Furthermore, neonatal rodent studies have shown that increased delay in initiation of 5h hypothermia is associated with decrease in protection [12], further strengthening the need for early intervention. However, the minimum protective hypothermia duration has not been optimised/established.

The aim of this work was to compare exposure to three different short periods of hypothermia, i.e. 1h, 1.5h and 2h, following the modified Rice-Vannucci model of neonatal mouse HI [13–15] in C57/Bl6 mice, and to assess subsequent levels of anatomical and functional protection.

## Materials and methods

### HI insult

All animal experiments and care protocols were carried out according to the UK Animals (Scientific Procedures) Act 1986 and approved by the UK Home Office and the UCL Animal Welfare and Ethical Review Board. The ARRIVE guidelines were followed. All experiments involved postnatal day 7 (P7) C57/Bl6 mice bred in house.

The mouse brain at postnatal day 7 is equivalent to human brain late-preterm/near term [16], and therefore relevant for studies of HIE, as it presents phenotypical similarities to the grey and white matter injury observed in human foetuses, i.e. tissue loss, cell-death, microglia-mediated immune response and astrogliosis as well as alteration in neurobehavioral performance [7].

The surgical procedures were performed as previously described [13–15,17]. Briefly, male and female littermate P7 mice were anaesthetised with isofluorane (5% induction, 1.5% maintenance). The left common carotid artery was permanently occluded with 8/0 polypropylene suture and the wound closed with tissue glue. The mice recovered at 36°C and were returned to the dam for 2h. The pups were then placed in a hypoxia chamber and exposed to humidified 8% oxygen/92% nitrogen (3L/min) at 36°C for 60min, resulting in moderate brain damage [14,15].

### Induced hypothermia

**Fig 1:**
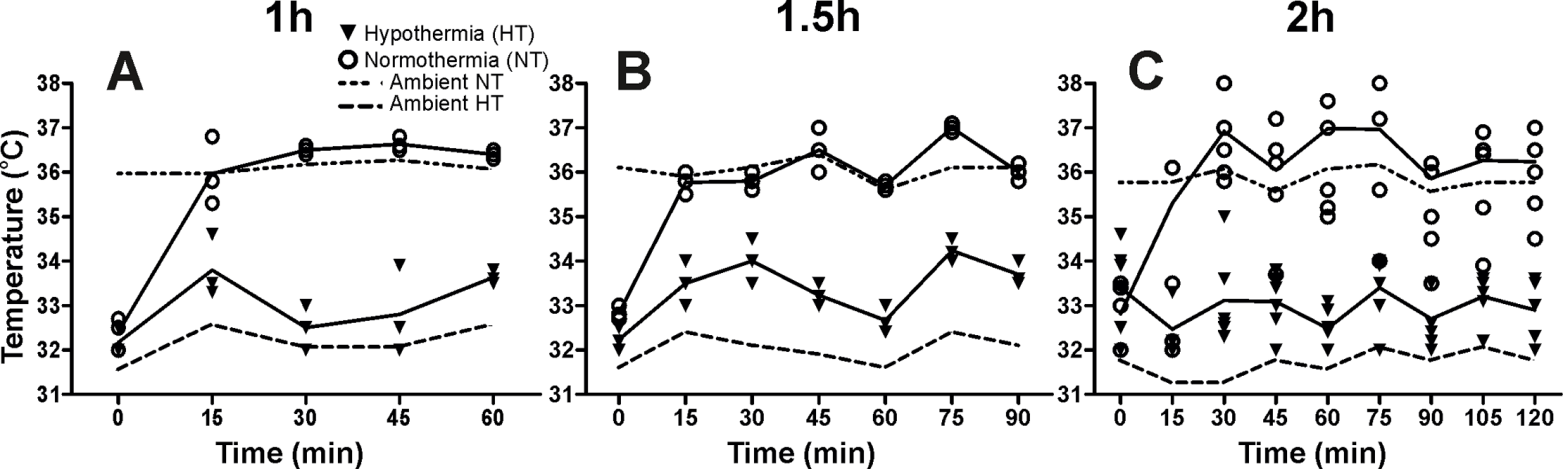
Summary diagram of the average temperature during (A) 1h, (B) 1.5h and (C) 2h of hypothermia/normothermia induced at 20min after neonatal HI (individual values and mean±SEM). The temperature for the hypothermia/normothermia groups was calculated as an average of the oral temperature for 3 different animals per group per time point, measured with a neonatal digital pacifier thermometer. The graphs show the mean ambient temperature for the hypothermia (HT) and normothermia (NT) chambers. The target temperature for normothermic animals was 36°C and for the hypothermic 33°C (**A**, **B, C,** HT n = 3 per time point, NT, n = 3 per time,).

Following exposure to 60min hypoxia the animals were returned to the dam for 20min. The animals were randomized to receive either hypothermia (1h n = 11, 1.5h n = 15, 2h n = 11) or normothermia (1h n = 10, 1.5h n = 12, 2h n = 11). The animals were exposed to ambient temperature of 32°C (hypothermia) or 36°C (normothermia) in a circulation water bath, in order to achieve body temperature of 33.5°C (hypothermia) or 36.5°C (normothermia) (Fig 1). We simulated exposure to therapeutic hypothermia, which is widely used in the clinical practice for treatment of HIE. The temperature of three animals per group was measured with a pacifier thermometer (Philipps Avent SCH540) every 15min and then averaged to represent the overall group temperature per time point (Fig 1, individual values and mean±SEM). The accuracy of the oral temperature assessment was validated with infrared thermal camera Optris PI 160 (BRSL, UK) which registers body core temperature, showing no significant differences between the values measured with a pacifier thermometer and the infrared thermal camera (data not shown).

In a separate control experiment the temperature of animals (n = 5) exposed to a 60min HI insult was measured before and for 48h after HI. This was to assess whether the temperature changes registered in the therapeutic hypothermia set-up were due to the treatment or to natural nesting surrounding (S1 Fig, individual values and mean±SEM) and whether the animals become and remain hypothermic in the nest following HI. We also wanted to eliminate the possibility of secondary hypothermia modulated by injury. The animals became naturally hypothermic in the nest for 1h after HI insult (S1 Fig, [18]), but from 2h till 48h the temperature was similar to start of experiment (before occlusion).

### Tissue sample preparation

The animals were sacrificed at 48h post-HI for histopathology. The time point with the highest level of widespread neuronal caspase-3 expression within the acute injury phase occurs 48h post-HI [19]. The mice received a terminal intraperitoneal injection of pentobarbitone and were perfused with 30mL 4% paraformaldehyde in 0.1M phosphate buffer (PB). The brains were removed, post fixed in 4% paraformaldehyde/0.1M PB for 1h at 4°C, and cryoprotected in 30% sucrose/PB solution for 24h as previously described [15,17]. The brains were then frozen on dry ice, cut on a cryostat into sequential 40-um sections and stored at −80°C until required.

### Immunohistochemistry and histological analysis

Five sections from each brain (400um apart), were rehydrated in distilled water and stained using immunohistochemistry as previously described [20]. Briefly, the sections were incubated overnight with rat anti-CD11b αM integrin subunit (1:5000, Serotec, UK) or rabbit polyclonal anti-GFAP (1:6000, DAKO, UK) primary antibodies, for 1h with biotinylated goat anti-rabbit or -rat (1:100, Vector, UK) secondary antibody, followed by incubation with Avidin-Biotinylated horseradish peroxidase Complex (Vector, UK) and visualisation with diaminobenzidine/H_2_O_2_ (Fisher Scientific, UK).

Five further sections from each brain with the same spacing were stained using Terminal transferase mediated d-UTP nick end labelling (TUNEL) (Roche, UK). The staining procedure followed the manufacturer protocol with Co/Ni enhancement.

Five more sections per brain with the same spacing were stained with cresyl violet (Nissl).

All sections belonging to the same hypothermia setting were stained simultaneously to avoid differences in intensity.

### Infarct volume measurement

The cresyl violet stained sections were scanned and imported into Optimas 6.5 image analysis software (Media Cybernetics). The areas of intact staining in the cortex, pyriform cortex, hippocampus, striatum, thalamus and external capsule were outlined and bilaterally measured. The percentage tissue loss was then calculated by converting the measured injured and uninjured areas into square millimetres and then transformed to a volume through multiplication by 400 μm. The sum of these volumes was then used to calculate the percentage of surviving brain tissue as ipsilateral/contralateral × 100 [13].

In some brains the natural asymmetry between the contralateral and ipsilateral hemispheres may result in the healthy experimental ipsilateral hemisphere before the HI insult being larger than the control one. Therefore, even though the HI insult would have caused shrinkage of the ipsilateral hemisphere, that would not have been enough to overcome that difference thus producing a negative tissue loss value.

### TUNEL

TUNEL positive cell death was assessed at 48h following HI through bilateral counting of the number of positive cells in three different optical fields at x20 magnification in the cortex, pyriform cortex, hippocampus, striatum, thalamus and external capsule. The counts were then averaged per animal and per group [15].

### AlphaM score

Immunohistochemistry for αMβ2 integrin as an early microglial activation marker [14,15,17,21–23], was performed as previously described [15,17]. Strong microglial immunoreactivity for the aMβ2 integrin is localised on the phagocytic microglial nodules which intensely stain with IBA-1 [23]. Increased number of αMβ2 integrin positive cells corresponds to higher levels of HI injury as increase in IBA-1 and αMβ2 is shown in the rodent model as early as 24h post HI [24]. We have previously shown that high levels of αMβ2 integrin immunoreactivity positively correlate with TUNEL+ cell death [13–15,17]. Furthermore, ramified microglia when activated undergo morphological changes to amoeboid form, and then become active phagocytes [25], Thus, higher levels of αMβ2 immunoreactivity were associated with increased tissue damage. Increased production of αMβ2 integrin is associated with microglial expansion and activation and is concurrent with morphological changes [24,26]. Therefore, semi-quantitative scores (Table 1) reflecting the intensity of the aMβ2 integrin immunoreactivity, were allocated to each brain region—cortex, pyriform cortex, hippocampus, striatum, thalamus and external capsule—by an observer blinded to the treatment groups.

**Table 1:** Semi-quantitative scores for αMβ2 integrin immunoreactivity.

Score	Microglial Appearance
0	**No activation**
**1**	**Focal microglial activation**
**2**	**Mild phagocytic activation affecting less than 50% of the region**
**3**	**Phagocytic activation affecting more than 50% of the region**
**4**	**Total phagocytic activation**

### GFAP optical luminosity

The intensity of the GFAP staining in the tissue was assessed using optical luminosity values [15,17]. Images for the ipsilateral hemisphere were captured with a Sony AVT-Horn 3CCD colour video camera (24bit RGB, 760x570 pixel resolution) in three different optical fields in cortex, pyriform cortex, hippocampus, striatum, thalamus and external capsule, as well as the surrounding glass at x20 magnification. We used Optimas 6.5 software to obtain the mean and standard deviation (SD) for optical luminosity values (OLV). SD was subtracted from the mean for each image and the resulting value was subtracted from the values acquired for the surrounding glass [20].

### Behavioural assessment

In a separate set of experiments behavioural tests were performed in a quiet experimental room between 3 and 5pm, under normal light conditions [27,28]. The order of testing of the animals within the litters was randomly assigned. We evaluated reflexes at P9 and P14, and performed behavioural test of motor performance at P21, respectively 2, 7 and 14 days after the HI insult and 2h of hypothermia treatment.

A Negative geotaxis is a method widely used for assessment of labyrinthine reflex, strength and co-ordination of neonatal mice [29]. Briefly, the pups are placed facing down on a platform inclined at 45° and the time for the pups to change their orientation upwards is measured. The animals were given 2 trials of 30s 10min apart, and the average between the two was used. Time exceeding 30s was considered a failure and was assigned as 30s and used in the calculations as such. The time was then averaged per group and per time point.
B The slipping test used is a modification of the balance beam test [30] used for evaluation of motor balance and co-ordination. This test detects motor deficits resulting from central nervous system lesions, as well as aging and genetic and pharmacological interventions [30]. The apparatus consists of a metal grid 50cm in length, placed about 20cm above a table top between the housing cage and a new clean cage. The starting point is the clean cage and the finish consisted of the housing nesting cage. The animals were allowed to walk freely on the grid for 1min. The task was recorded and the videos were reviewed by two team members blinded to the mouse groups. The number of missed steps was counted and presented as a percentage of the total number of steps for each animal. The results were then averaged per group.

### Statistics

The number of animals/group is estimated with power calculations based on detection of a 30% difference (p<0.05 and power >0.8) and a standard deviation as in earlier studies with a similar design [22]. All histological data was tested for normal distribution and corrected with logarithmic transformation if not normally distributed. Statistical significance in the histological analysis was assessed through repeated testing using Mixed Linear Model with SPSS 13.0 software, treating region as the repeated measure. For each outcome six regions of the brain were examined. It is likely that with repeated measures such as these the observations from a single subject are correlated, therefore the first stage of the analysis included the observations from all the regions tested in a single mixed model with a random subject effect, to produce an estimate of the treatment effect and associated inference that accounts for the correlations in the data arising from the repeated measures. Further post-hoc Student t-tests were carried out to assess evidence for subregional differences, p<0.05. For each outcome, the overall effect from the mixed linear model is reported, followed by the results from the individual regional t-tests. All data is presented as individual values and median ± interquartile bars.

Statistical significance in the negative geotaxis behavioural testing was assessed through one–way ANOVA with Sidak’s multiple comparison test (for P14) or Kruskal Wallis with Dunn’s multiple comparison test (for P9), for normal or non-normal distribution respectively. Normal distribution of the data was assessed using Shapiro-Wilk normality test. We used Mann-Whitney test to assess significance in the case of slipping test analysis at P21.

## Results

### Modelling viability of short-term duration hypothermia

**Fig 2:**
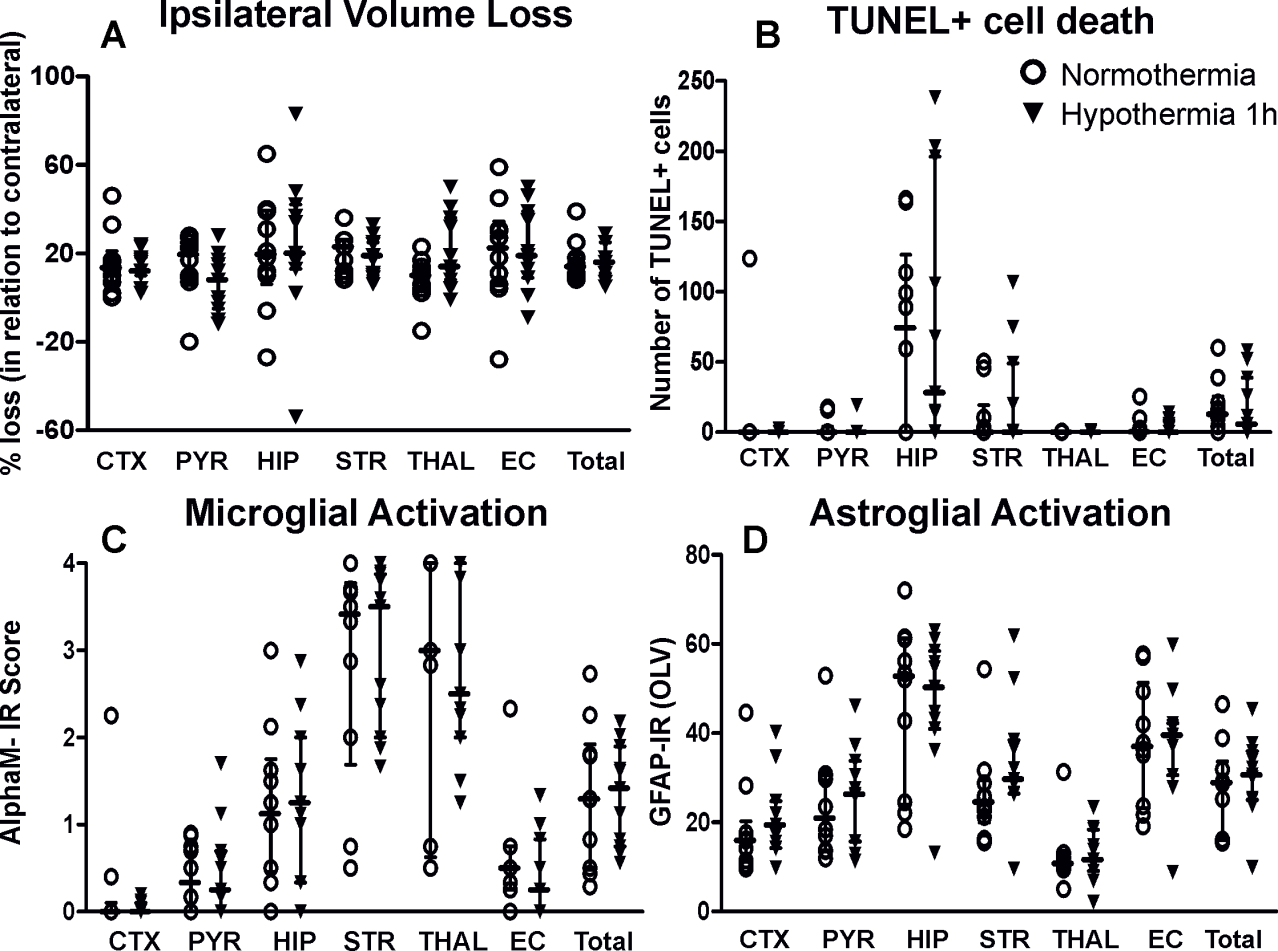
Exposure to 1h hypothermia/normothermia does not provide neuroprotection following neonatal HI. A) Ipsilateral brain tissue volume loss was not significantly affected in any of the examined brain regions in the hypothermia treated animals compared to normothermia treated controls. B) The number of TUNEL+ dying cells (per 20x eye-field) at 48h following HI, was not decreased in the hypothermia treated group compared to normothermia treated littermate controls. C) Ipsilateral microglial activation score (individual values and median ± interquartile bars) based on the αM integrin immunoreactivity was not decreased in any of the examined regions and overall was not significantly affected in the hypothermia treated group (n = 11) compared to the normothermia treated littermate controls (n = 10). D) Ipsilateral reactive astrogliosis (GFAP immunoreactivity in OLV) was not significantly affected in any of the examined brain regions in the hypothermia treated group compared to normothermia treated littermate controls. ***Abbreviations*:** CTX–cerebral isocortex, PYR–pyriform cortex, HIP–hippocampus, STR–striatum, Thal–thalamus, EC–external capsule, IR–immunoreactivity.

To assess the biological impact of different short periods of hypothermia, i.e. 1h, 1.5h and 2h, following neonatal HI, we subjected P7 mice to either hypothermia (33.5°C) or normothermia (36°C) at 20min post 60min HI. Fig 1 represents the average animal temperature measured with a pacifier thermometer every 15min during the altered temperature exposure: 1h (Fig 1A), 1.5h (Fig 1B) and 2h (Fig 1C), and the respective ambient temperatures. As at P7 thermoregulation is highly immature, the animals become slightly hypothermic immediately and for approximately 1h after the insult once returned to the nest [31] compared to normal adult values (S1 Fig, [18]). The temperatures return to normal values within 2h post HI. This can explain the slightly hypothermic temperature values observed at the beginning of treatment.

### 2h hypothermia prevents brain injury 48h after HI

To evaluate the effect of exposure to short period of hypothermia (1h) on neonatal HI brain damage, we examined the effects on volume loss (Fig 2A), TUNEL+ cell death (Fig 2B), early microglial activation (Fig 2C), and reactive GFAP+ astrogliosis (Fig 2D) at 48h following HI. Fig 2 shows that exposure to 1h of hypothermia did not affect any of the markers of brain damage, compared to normothermia treated littermate controls.

**Fig 3:**
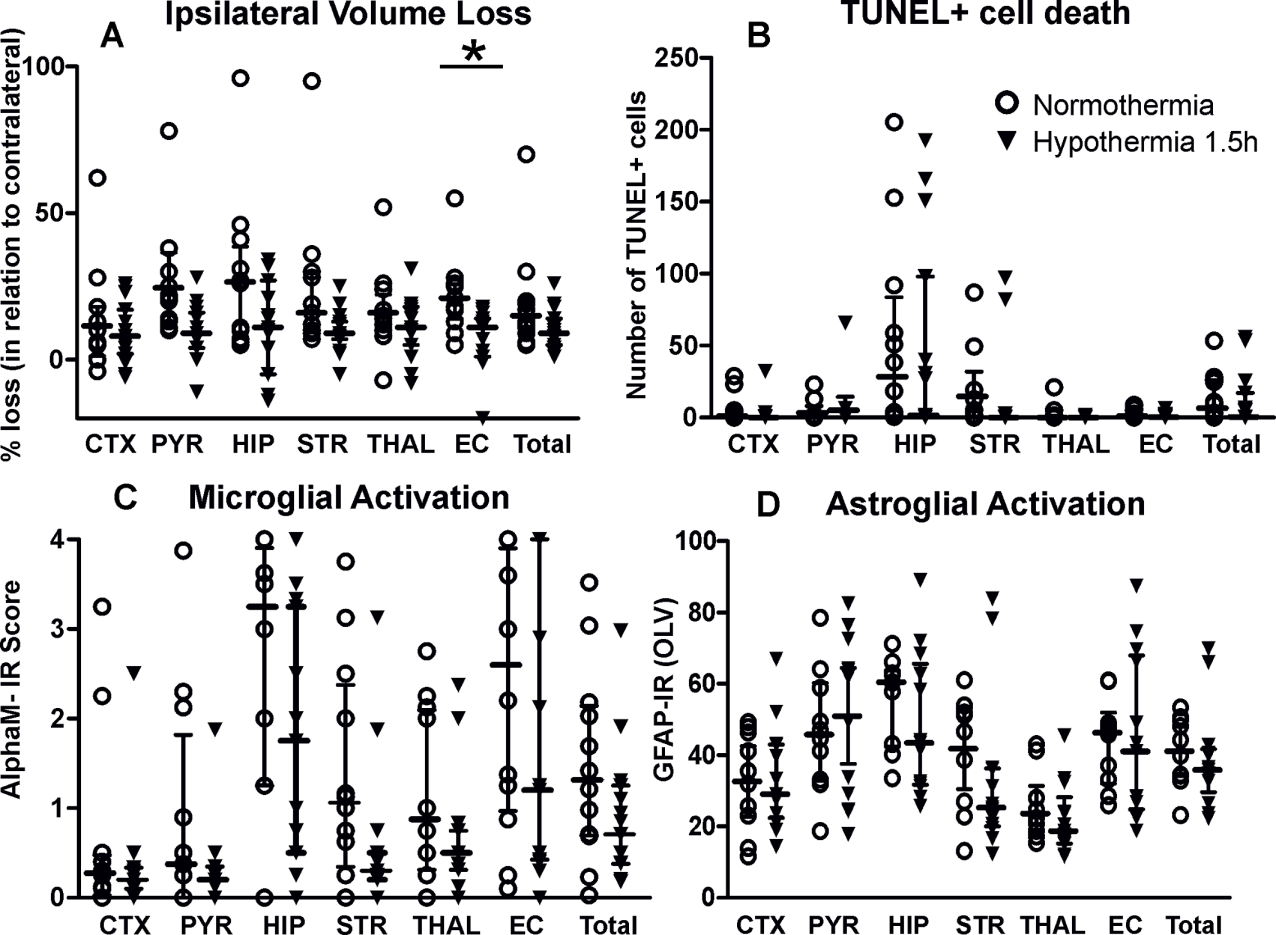
Exposure to 1.5h hypothermia/normothermia reduces ipsilateral brain tissue volume loss following neonatal HI. A) 1.5h of hypothermia significantly reduced ipsilateral brain tissue volume loss (individual values and median ± interquartile bars) compared to normothermia treated controls, with significant individual decrease (t-test) in external capsule (p = 0.0125) and consistent lower tissue loss values in the rest of the examined regions. Mixed Linear Model treating region as a repeated measure revealed p = 0.033. B) The number of TUNEL+ dying cells (per 20x eye-field) at 48h following HI, was not decreased in the 1.5h hypothermia treated group compared to normothermia treated littermate controls. C) Ipsilateral microglial activation score (individual values and median ± interquartile bars) based on the αM integrin immunoreactivity was overall not significantly affected despite the consistent lower average score in all of the examined regions in the 1.5h hypothermia treated group (n = 15) compared to the normothermia treated littermate controls (n = 12). D) Ipsilateral reactive astrogliosis (GFAP immunoreactivity in OLV) was not significantly affected in any of the examined brain regions in the 1.5h hypothermia treated group compared to normothermia treated littermate controls. ***Abbreviations*:** CTX–cerebral isocortex, PYR–pyriform cortex, HIP–hippocampus, STR–striatum, Thal–thalamus, EC–external capsule, IR–immunoreactivity.

Compared to animals exposed to 1.5h of normothermia, exposure to the same period of hypothermia (Fig 3) resulted in overall significant decrease of tissue loss values (Fig 3A, Mixed Linear Model treating region as a repeated measure p = 0.033) with individual significant decrease in external capsule (t-test, p<0.05). Exposure to 1.5h of hypothermia did not affect the levels of TUNEL+ cell death (Fig 3B), and showed a trend towards reduced microglial activation based on αMβ2 integrin immunoreactivity but did not reach statistical significance overall (Mixed Linear Model treating region as a repeated measure p = 0.332) or in any of the examined brain regions (Fig 3C). There were no changes in reactive astrogliosis (GFAP-immunoreactivity, Fig 3D) compared to littermate control animals subjected to the same period of normothermia.

**Fig 4:**
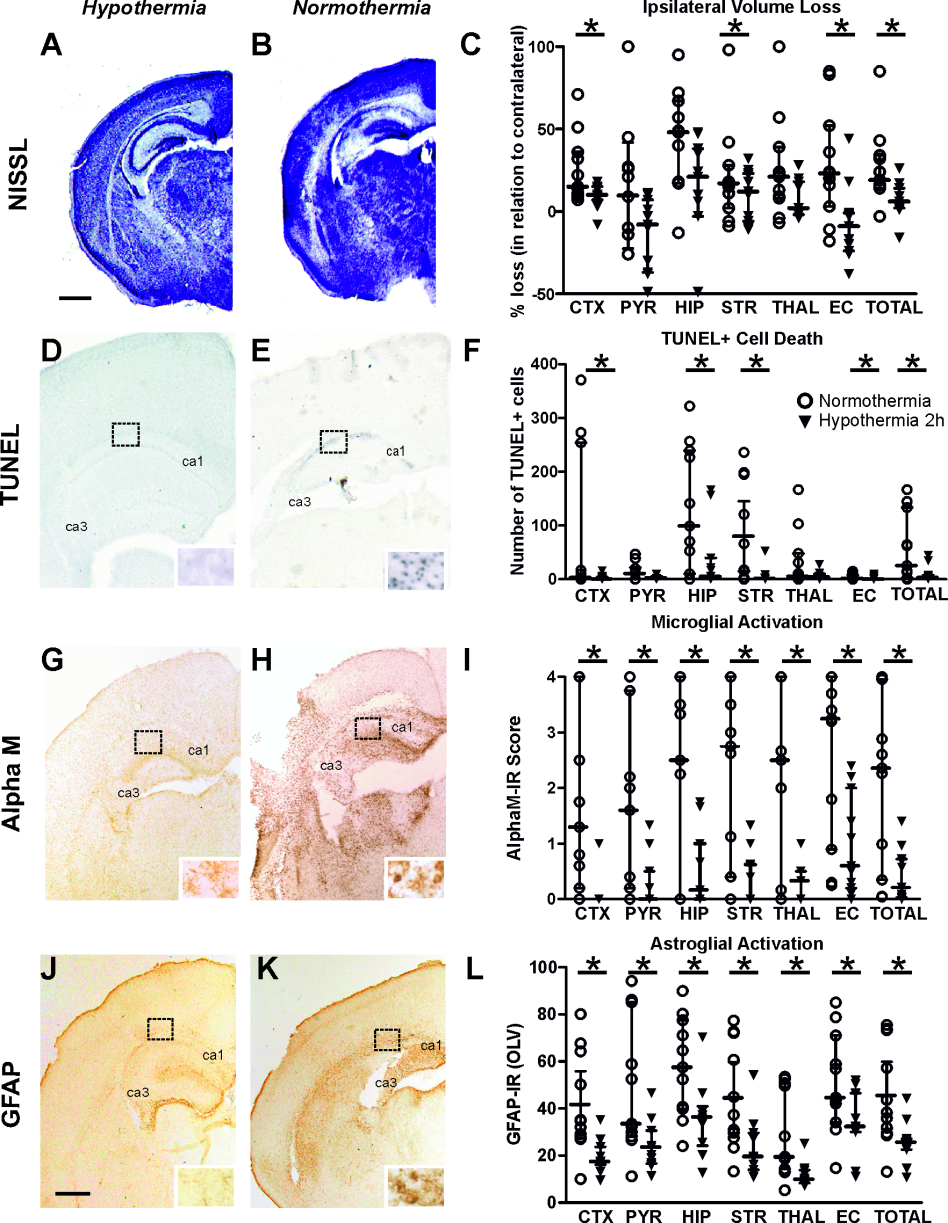
2h hypothermia/normothermia reduces microglial activation, reactive astrogliosis, TUNEL+ cell death and brain tissue loss following neonatal HI. **A-C:** Ipsilateral Nissl staining (Cresyl-Violet, at rostral parietal level) of 2h hypothermia (A) and normothermia (B) animals and ipsilateral volume loss quantification (C) 48h post-HI. 2h hypothermia reduced volume loss (individual values and median ± interquartile bars) compared to normothermia controls, with significant, individual decrease (t-test) in cortex (p = 0.020), striatum (p = 0.049), external capsule (p = 0.005) and total volume loss (p = 0.040). MLM p = 0.038. **D-F:** TUNEL+ staining at 48h post-HI–Ipsilateral overview in 2h hypothermia (D) and normothermia (E) animals and quantification (F) (number of TUNEL+ cells per 20x eye-field, individual values and median ± interquartile bars). The normothermia group showed typical pyknotic nuclei of the TUNEL+ cells (E-insert, hippocampus) while the hypothermia group was lacking such cells (D). 2h hypothermia significantly reduced TUNEL+ cell death with significant individual decrease (t-test) in cortex (p = 0.032), hippocampus (p = 0.031), striatum (p = 0.022), external capsule (p = 0.039) and total TUNEL+ cell death (p = 0.024). MLM p = 0.024. **G-I:** αM+ microglia–Ipsilateral overview in hypothermia (G) and normothermia (H) animals and ipsilateral αM microglial activation score (I, individual values and median ± interquartile bars). Note the phagocytic morphology of the cells in the normothermia group (H-insert, hippocampus), compared to the ramified phenotype in 2h hypothermia treated brains (G-insert). 2h hypothermia reduced αM+ microglial activation across all 6 examined regions, with significant, individual decrease (t-test) in isocortex (p = 0.004), pyriform cortex (p = 0.007), hippocampus (p = 0.003), striatum (p = 0.001), thalamus (p = 0.004), external capsule (p = 0.006) and total microglial activation (p = 0.002). Mixed Linear Model treating region as a repeated measure (MLM) p = 0.002. Hypothermia (n = 11) and Normothermia (n = 11) in all assessments. **J-L**: GFAP immunoreactivity at 48h - Ipsilateral overview in 2h hypothermia (J) and normothermia (K) animals and ipsilateral quantification (L) in optical luminosity values (OLV, individual values and median ± interquartile bars). J and K inserts: higher magnifications of rostro-parietal isocortex. 2h hypothermia reduced astrogliosis, with significant, individual decrease (t-test) in cortex (p = 0.006), pyriform cortex (p = 0.016), hippocampus (p = 0.015), striatum (p = 0.018), thalamus (p = 0.010) external capsule (p = 0.047) and total GFAP immunoreactivity (p = 0.013). MLM p = 0.009. ***Abbreviations*:** CTX–cerebral isocortex, PYR–pyriform cortex, HIP–hippocampus, STR–striatum, Thal–thalamus, EC–external capsule, IR–immunoreactivity. *p<0.05 ***Scale bars*: A, B, D, E, G, H = 600um; inserts = 30um; J, K = 700um**.

As shown in Fig 4A–4C, exposure to 2h of hypothermia treatment significantly reduced forebrain tissue loss assessed through the ratio of the volume of the ipsilateral versus the non-occluded contralateral hemisphere. Fig 4B shows infarct in mid-lateral cortex and hippocampus of the normothermia treated animal and it’s sparing in the 2h hypothermia littermate (Fig 4A). Quantification of ipsilateral volume loss (Fig 4C) revealed strong decrease across the different forebrain regions (Mixed Linear Model treating region as a repeated measure, p = 0.038), with individual significant decrease of 60–95% in cortex, striatum, external capsule and total forebrain area (t-test, p<0.05).

We next examined the biological impact of 2h hypothermia treatment on TUNEL+ cell death. Fig 4D–4F show that 2h hypothermia treatment markedly reduced the number of TUNEL+ cells compared to normothermia treated littermate control (Fig 4D). The TUNEL+ cells displayed the typical pyknotic nuclear morphology (Fig 4E-insert, ipsilateral hippocampus normothermia). The hypothermia induced reduction was observed across all studied forebrain regions (Mixed Linear Model, treating region as a repeated measure, p = 0.024). Individually, all 6 regions revealed a significant, 70–90% decrease in the number of TUNEL+ cells in the 2h hypothermia treated animals compared to their normothermic littermates (Fig 4F, t-test, p<0.05).

Microglial activation score was significantly reduced (Fig 4I) based on the αMβ2 integrin immunoreactivity (Fig 4G & 4H). Regional assessment shown in Fig 4I revealed a reduction in activation score in the 2h hypothermia treated group (Mixed Linear Model treating region as a repeated measure p = 0.002), with a significant decrease of 60–90% in all studied individual ipsilateral brain regions (t-test, p<0.05). While the microglial cells in the control normothermia treated group revealed the round, phagocytic phenotype shown in the insert of Fig 4B, the microglial cells detected in the 2h hypothermia treated group mainly displayed resting to activated, but still ramified morphology (Fig 4G-insert).

In addition to microglial activation, 2h hypothermia also decreased HI-induced ipsilateral reactive astrogliosis (Fig 4J–4L). Compared to normothermia treated control animals, their 2h hypothermia treated littermates revealed less overall GFAP-immunoreactivity (Fig 4L) with substantially reduced and more spotty areas of GFAP+ astroglial processes (Fig 4J & 4K). Assessment across the different forebrain regions through Mixed Linear Model treating region as a repeated measure revealed a clear decrease of GFAP immunoreactivity (p = 0.009), with individual significant decrease of 30–60% in all studied regions (t-test, p<0.05).

### 2h hypothermia results in functional protection 7 days after HI

**Fig 5:**
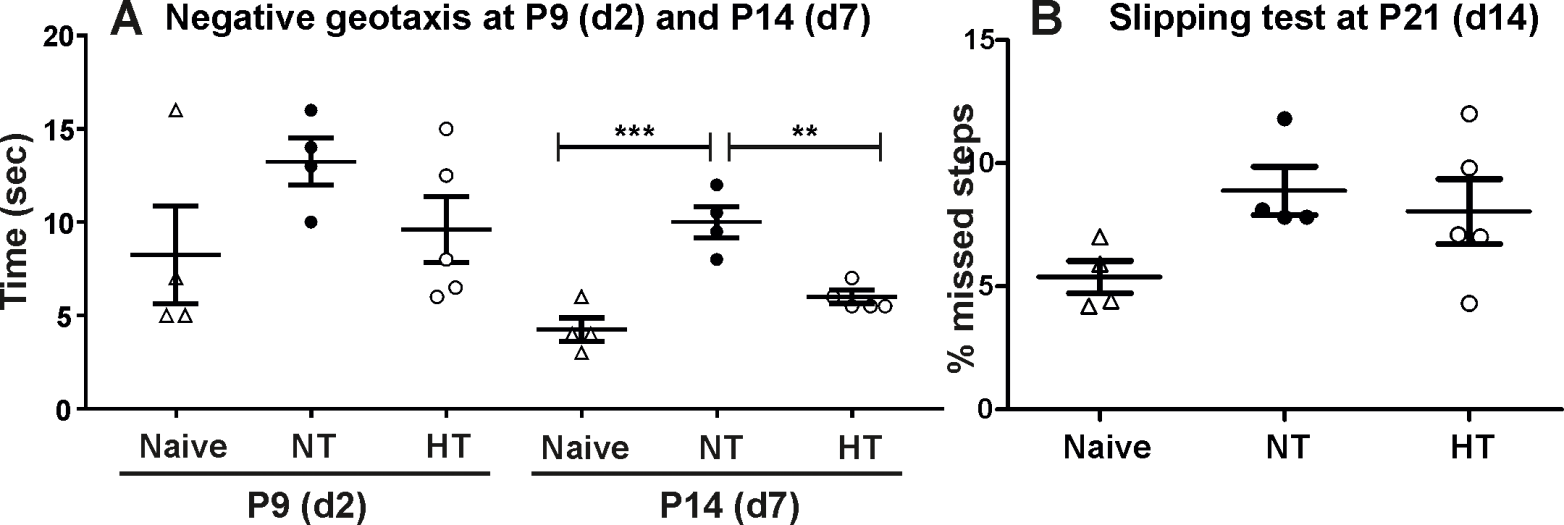
Exposure to 2h hypothermia/normothermia reduces the time required to change orientation in negative geotaxis following neonatal HI, but has no effect in slipping test. A) Negative geotaxis at 48h post-HI (postnatal day 9) and 7 days (postnatal day 14). No differences were observed at postnatal day 9 between naïve, normothermia and 2h hypothermia treated animals (Kruskal Wallis test with Dunn’s multiple comparison test). At 7 days post-HI normothermia treatment significantly increased the time needed for change of orientation when compared to naïve controls (p = 0.0004). 2h hypothermia treatment significantly reduced the time necessary for change of orientation compared to normothermia treated littermates (p = 0.005; one–way ANOVA with Sidak’s multiple comparison test). Hypothermia (n = 5), Naïve (n = 4) and Normothermia (n = 4) in all assessments, B) 2h of hypothermia treatment had no effect on the number of missed steps (slipping test) at 14 days (postnatal day 21) post-HI (Mann-Whitney test).

Assessment of behavioural changes through negative geotaxis at 48h post-HI (postnatal day 9) did not show differences between 2h hypothermia treated animals and their normothermia treated or naïve littermate controls (Fig 5A). However assessment of the same animals and groups at 7 days post-HI (postnatal day 14) showed significant increase in the time required for the normothermia treated pups to change their orientation upwards compared to naïve littermate controls (Fig 5A). Interestingly, 2h of hypothermia treatment significantly reduced the time for change of orientation compared to normothermic littermate controls with levels close to the ones observed in naïve animals (Fig 5A).

Assessment of motor balance and co-ordination through slipping test at day 14 post-HI (P21) of 2h hypothermia treated animals and normothermic and naïve littermate controls did not show differences between the groups (Fig 5B).

## Discussion

Compelling evidence from animal studies and randomised controlled clinical trials suggests that mild therapeutic hypothermia in late preterm and term new-born infants with HIE significantly improves survival and disability [32,33]. Consequently, therapeutic hypothermia is now standard care for infants with moderate to severe HIE [33]. However about 40% of hypothermia treated children have neurodevelopmental incapacities [34,35]. This urges the necessity to develop combined treatment including effective hypothermia protocols and other known neuroprotective agents [33] such as erythropoietin [36] and melatonin [37]. Clinically, mild induced hypothermia is initiated within 6h of birth and lasting for 72h. However, this is not the case in basic and translational animal models, where cooling is initiated and maintained within varied periods after HI. Large translational models, such as the newborn piglet, perform cooling 2-26h post-HI, with visible neuroprotective effect [35,37,38]. They also demonstrate that depth of cooling higher than 3.5 degrees does not improve cell survival outcome, and may even be deleterious [35,38]. Another advantage of preclinical models is the continuous physiological monitoring [37], similar to clinical setup.

However, as further research to systematically test combined neuroprotective treatments with hypothermia is critical and experiments in large animal models are expensive and time consuming, new approaches are easier to test and develop or exclude in the well-established rodent Rice-Vannuci model.

Different mouse strains used in the Rice-Vannucci model show different susceptibility to the HI-induced brain damage [17,39,40], however the C57/Bl6 strain is considered and remains one of the most suitable and widely used inbred strain in this model showing substantial brain damage following relatively short exposure (50-60min) to decreased oxygen environment. The pattern of damage in the C57/Bl6 neonatal mouse is also consistent with the HI-induced brain damage observed in humans. Furthermore, the wide availability of transgenic mice bred in the C57/Bl6 background make it very relevant for understanding mechanisms and pathways involved in injury and protection. Several previous studies in the mouse model have used hypothermia alone [8,9] or in combination with other agents [41] to provide neuroprotection, but have suggested exposure to periods of hypothermia varying between 3h [41,42], 4h, 5h [8,9], and 6h [43] and have demonstrated varying degrees of neuroprotection. Such variability of results and protocols indicate the need for further research to improve outcome. The short-term duration of therapeutic hypothermia used in the rodent HI model is not directly translational to the clinical settings, however, it has shown applicability for use in larger translational models leading to clinical trials [44–46].

To the best of our knowledge the minimal time to provide neuroprotection in the Rice-Vannucci model and the C57/Bl6 mouse strain, has never been stated. A unified protocol providing neuroprotection similar to the observations from the clinical hypothermia procedures, with minimal time for exposure to hypothermia in the P7 neonatal C57/Bl6 mouse HI model would allow easier experimental design involving multiple therapies, which are more tolerant for the experimental animals and less time consuming for the researcher.

Despite the variability in the severity of the insult between the three hypothermia settings, our assessment of HI brain damage based on infarct volume, TUNEL, alpha M and GFAP suggests moderate brain injury. Exposure to 1h and 1.5h of hypothermia following 60min HI does not reduce brain damage. However, exposure to 2h of hypothermia results in significant short term neuroprotection in the P7 neonatal C57/Bl6 mouse demonstrated histologically at 48h (48h, Fig 4), as well as behaviourally at 7 days (Fig 5). Interestingly, the data from the negative geotaxis show no differences in the shorter period (P9, Fig 5A). This suggests that this time point may not be ideal to perform this test, and too short to observe changes in behaviour post-HI, as seen by the lack of difference when compared to naïve mice. However, the improved strength and co-ordination observed through negative geotaxis at P14 confirms the moderately long term neuroprotective effects provided by 2h hypothermia treatment. The lack of effect of the 2h hypothermic treatment on motor balance and co-ordination assessed through the slipping test 14d after HI is likely due to differences in sensitivity between the two methods of behavioural testing or could imply either endogenous regeneration in the normothermia group, or delayed injury processes in the HT group resulting in similar brain injury at P21. Therefore, we cannot exclude the possibility that 2h hypothermia alone may result in transient, rather than long term neuroprotection. This represents a limitation in our current study, where longer term survival beyond the neonatal period would enable confirmation whether the short (2h) hypothermia protection is sustained. The longer-term survival would also allow discrimination in neuroprotection between male and female mice, as studies have shown potential differences [9]. The differences between male and female mice were not taken into account, however our assessments were conducted within the neonatal period where sex differences would not have influenced the outcome [47]. Nevertheless our results are in line with the partially protective effects of hypothermia treatment detected in the clinical practice, where a high percentage of the hypothermia treated children still develop neurological disabilities.

The neonatal mouse model of HI-induced brain damage has high variability which is increased even more by the use of different mouse strains [17,39], the length of hypoxia and the susceptibility of the different brain regions to damage and protection [10,48]. However, as the C57/Bl6 mouse strain is the commonly used inbred mouse strain for neonatal HI studies, our data suggests that for the development of combined therapies, exposure of this strain to 2h of hypothermia after 60min HI would provide sufficient short term neuroprotection relevant to the clinical outcome.

## Supporting information

S1 Fig Temperature changes after neonatal HI. **T**he temperature of animals (n = 5) exposed to a 60min HI insult was measured before and for 48h after injury. The animals became naturally hypothermic in the nest immediately and for 1h after HI insult (33.8°C), but from 2h till 48h the temperature was similar to start of experiment (before occlusion).
(TIF)
