# Supplementary figures and images for: The duration of hypothermia affects short-term neuroprotection in a mouse model of neonatal hypoxic ischaemic injury
Source: PLoS One. 2018 Jul 3;13(7):e0199890. doi: 10.1371/journal.pone.0199890 (PMC6029790; doi:10.1371/journal.pone.0199890)

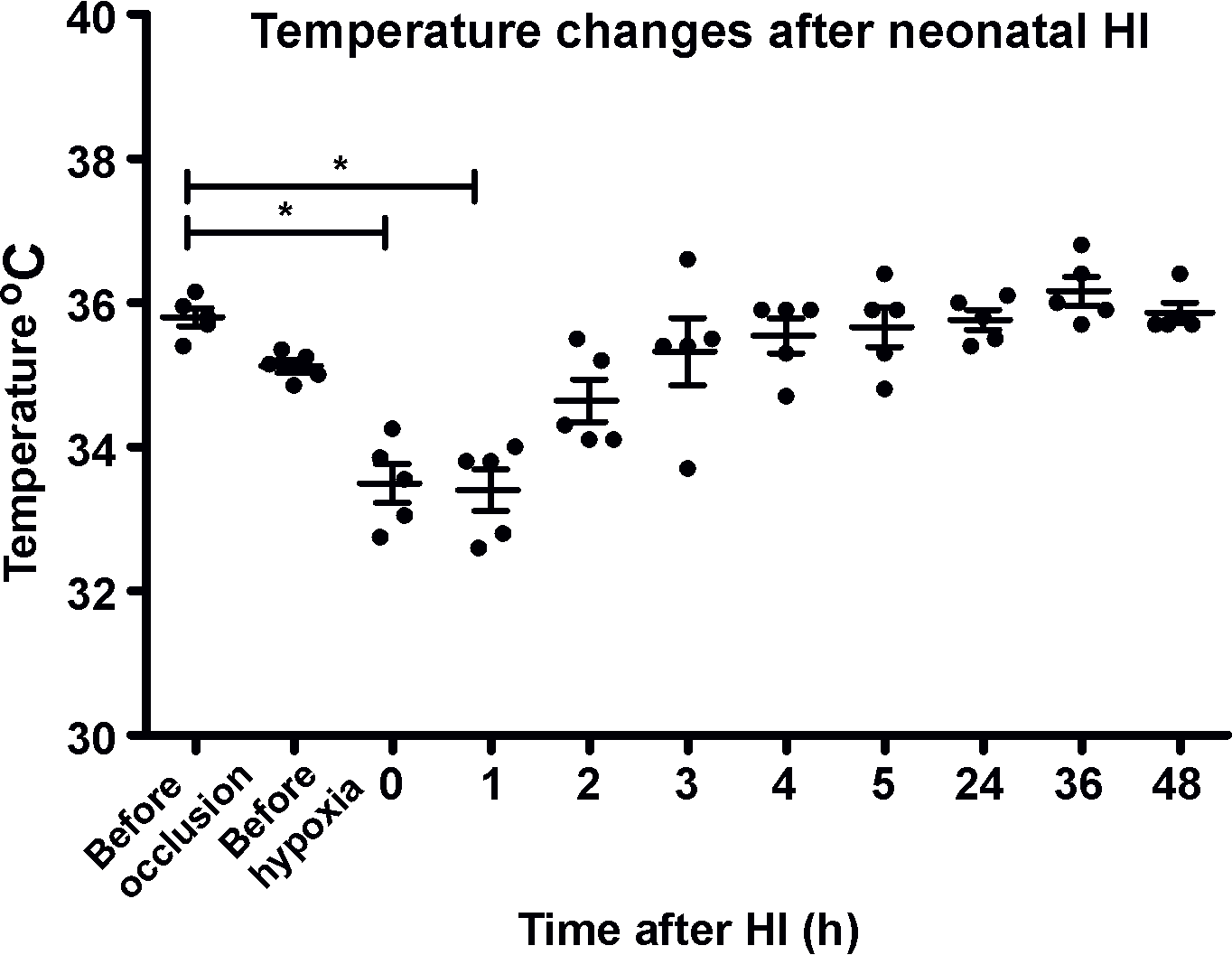

Supplement: S1 Fig — The temperature of animals (n = 5) exposed to a 60min HI insult was measured before and for 48h after injury. The animals became naturally hypothermic in the nest immediately and for 1h after HI insult (33.8°C), but from 2h till 48h the temperature was similar to start of experiment (before occlusion). (TIF) [file pone.0199890.s001.tif]
